# Supplementary material for: Dynamics of Neutralizing Antibody Responses Following Natural SARS-CoV-2 Infection and Correlation with Commercial Serologic Tests. A Reappraisal and Indirect Comparison with Vaccinated Subjects
Source: Viruses. 2021 Nov 22;13(11):2329. doi: 10.3390/v13112329 (PMC8621742; doi:10.3390/v13112329)
Supplement: Supplementary file 1 [file viruses-13-02329-s001.zip › viruses-1450450-supplementary.pdf]

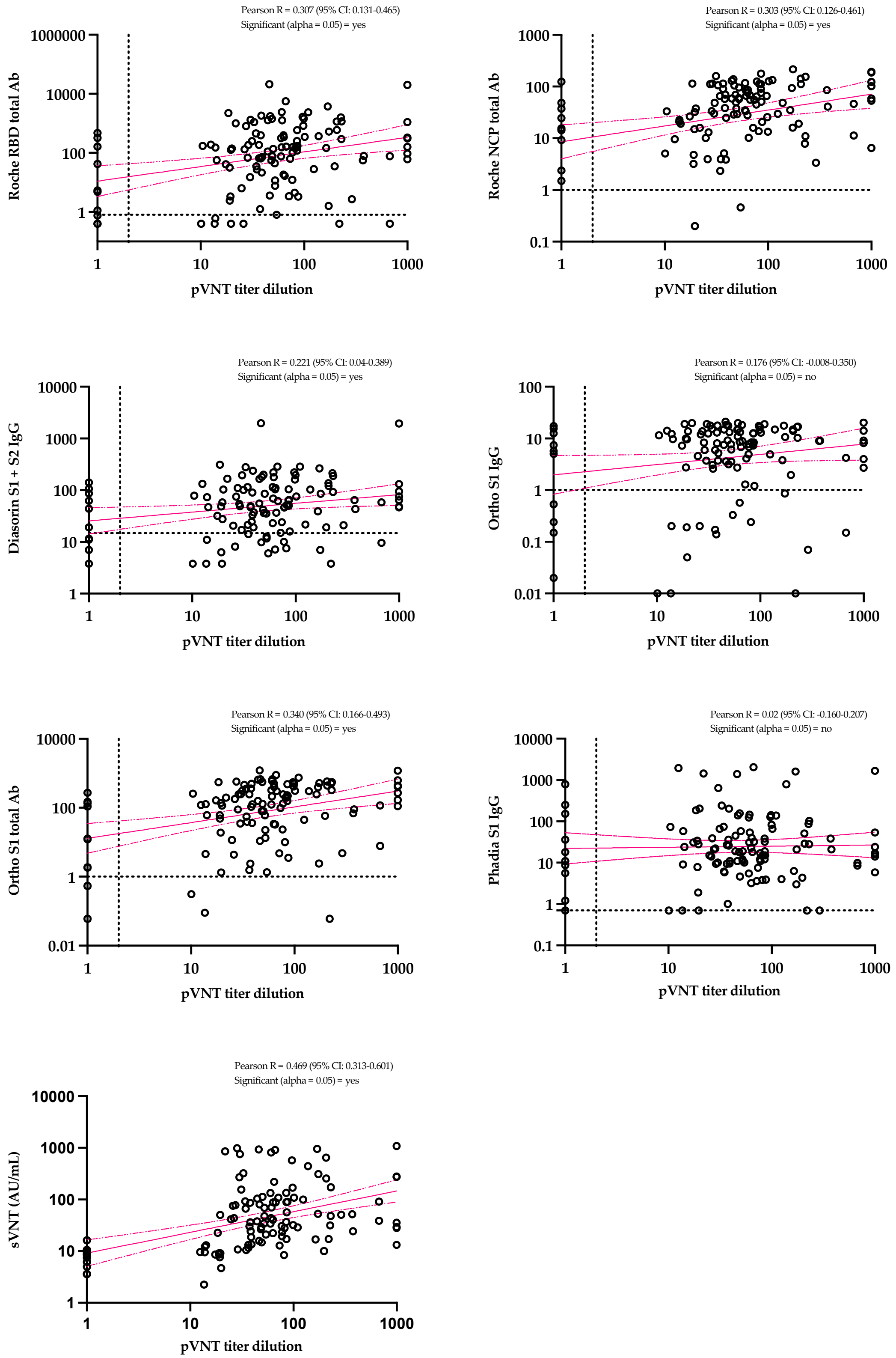

Figure S1. Head-to-head comparison of the pVNT to 6 non neutralizing immunoassays and the sVNT technique. Black dotted lines correspond to the positivity threshold of each assay.
